# Investigation of Normal Tissue Toxicity in Pulsed Low Dose Rate Radiotherapy

**DOI:** 10.3390/cancers17101701

**Published:** 2025-05-19

**Authors:** Shahabeddin M. Aslmarand, Troy Dos Santos, Dae-Myoung Yang, Dusica Cvetkovic, Lili Chen, C.-M. Charlie Ma

**Affiliations:** Department of Radiation Oncology, Fox Chase Cancer Center, Philadelphia, PA 19111, USAdaemyoung.yang@fccc.edu (D.-M.Y.);

**Keywords:** pulsed low dose rate radiotherapy, radiobiology, normal tissue toxicity

## Abstract

**Purpose:** Pulsed low dose rate radiotherapy (PLDR) is a radiotherapy approach expected to reduce normal tissue toxicity while maintaining equivalent tumor control as conventional radiotherapy (CRT). This preliminary study evaluates the effectiveness of PLDR in reducing normal tissue toxicity in vivo. **Materials and Methods:** In the initial phase, C57BL/6 mice underwent histological examination following single-fraction, total-body irradiation. Observations were conducted at 3 and 5 days post-treatment. Mice were divided into control, PLDR, and CRT groups, receiving varying doses ranging from 4 to 12 Gy. Building upon the histological findings, the second phase centered on whole-abdominal irradiation (WAI) and determining the lethal dose for WAI using CRT. Subsequently, this dose was applied in PLDR settings to compare survival rates and changes in body weight. The experiment was replicated to collect histology samples at 1-, 3-, 5-, 7-, and 9-day endpoints, enabling the assessment and comparison of tissue toxicity. Finally, exploration into PLDR’s lethal WAI dose was conducted. **Results:** Histology results showed the abdominal region as the main site of difference between PLDR and CRT, with both methods causing a dose-dependent increase in atrophy and hyperplasia. However, CRT led to higher tissue toxicity compared to PLDR. In the survival study, the fatal dose for WAI treatment was 18 Gy, with mice in the CRT group experiencing substantial weight loss and dying within 9–12 days post-treatment. In contrast, mice in the PLDR group, despite an initial weight loss, recovered their weight and survived. Histology results also showed that the PLDR group had less tissue toxicity. Furthermore, the fatal dose of WAI for PLDR was revealed to be 29 Gy, which is over 60% higher than the dose required for CRT, indicating a substantial difference in tolerance and potential safety margin provided by PLDR treatment. **Conclusions:** PLDR demonstrated a reduced normal toxicity compared to CRT, potentially beneficial in re-treatment scenarios or for tumors where CRT-induced toxicity limits tumor control, such as in liver cases.

## Simple Summary

Pulsed low-dose-rate radiotherapy (PLDR) is a radiotherapy technique believed to reduce damage to healthy tissues while maintaining cancer-killing efficacy comparable to conventional radiotherapy (CRT). In this study, we evaluated the effectiveness of PLDR in reducing normal tissue toxicity compared to CRT using a mouse model. Healthy mice were subjected to whole-abdominal irradiation with either CRT or PLDR. We assessed toxicity by monitoring survival rates and weight changes. The results demonstrate that PLDR induces significantly lower toxicity, with the lethal dose for PLDR being approximately 60% higher than that of CRT.

## 1. Introduction

There have been significant advancements in computer technology and radiation therapy (RT). These improvements have led to the development of advanced computer-controlled linear accelerators, complex multi-leaf collimators (MLCs), cutting-edge treatment planning systems, and sophisticated optimization techniques. Additionally, new imaging methods have allowed for incredibly precise treatment. Modern techniques like intensity-modulated radiotherapy (IMRT) and volumetric-modulated arc radiotherapy (VMAT) enable highly effective dose distribution. These technological advancements are improving tumor treatment, enhancing patients’ quality of life, and making treatments more cost-effective [1].

Even though recent technological advances have greatly improved our ability to control tumors using radiation therapy, there has not been as much progress in updating the underlying radiobiological theories. These theories guide how therapeutic radiation is used. A notable recent contribution in this field has been made by Song et al. [2]. They focus on two modern techniques: stereotactic radiosurgery (SRS) and stereotactic ablative radiation therapy (SABR) [3,4,5]. These methods typically deliver high radiation doses in one to five fractions. However, traditional radiobiology theories do not fully explain why these methods are effective. It is now believed that these techniques work not only by directly destroying tumor cells but also by damaging the blood vessels that supply the tumor, leading to tumor cell death due to a lack of blood supply. Furthermore, these methods might also help boost the body’s immune response against the tumor [6].

Another radiobiological phenomenon is low-dose hyper-radiosensitivity (HRS), which has been observed in the survival curves of many cell lines at low radiation doses [7,8]. One explanation for this effect is that DNA repair mechanisms are not immediately activated at very low doses, but are instead triggered only when a certain threshold dose, known as the transition dose, is reached [7,8,9]. This transition dose is cell-type dependent and is typically observed in the dose range of 0.2 Gy to 0.6 Gy.

Another explanation can be a dose rate effect. Several studies have demonstrated that dose rate can significantly influence tumor response. Interestingly, both high dose rates (e.g., >2 Gy/min) and very low dose rates (e.g., 0.1 Gy/min) have been associated with increased cell killing [10]. Despite this, delivering such very low dose rates is technically challenging using conventional linear accelerators.

This limitation has led to the development of a potential solution in the form of pulsed low dose rate radiotherapy (PLDR), which seeks to mimic the biological effects of continuous low dose rate delivery using a series of radiation pulses. In this treatment, the radiation dose has been broken up into smaller ‘pulses (subfractions)’ with time gaps in between. These pulses are a form of subfraction and should not be confused with the pulses of the linear accelerator used in other delivery techniques, such as flash therapy, etc. The length of these breaks is a key factor in determining the effectiveness of treatment. The idea behind this method is that tumor cells are more sensitive to damage when exposed to low-dose radiation pulses, while healthy tissues can effectively repair themselves from the damage caused by such low doses [8,11,12].

PLDR is especially promising for treating tumors that are close to or in contact with vital normal tissues. It offers a way to target the tumor while allowing the surrounding healthy tissue to heal. This approach could be particularly useful for treating tumors near areas that have already been treated and are close to reaching their safe radiation limit. The potential of PLDR to balance treatment effectiveness with tissue safety warrants a detailed study of its effects on both tumors and normal tissues. There is a relatively large body of evidence on the effectiveness of tumor control that we will briefly review here.

Tomé and Howard [13] performed an in vitro study of a PLDR scheme in human glioma. They have shown that a fraction size of 0.2 Gy delivered in a pulsed fashion over ten subfractions reduced the resulting surviving fraction for four out of the five cell lines used in their modeling that exhibited low-dose hyper-radiosensitivity (HRS) in vitro. The tumor control probability was improved for all cell lines with the pulsed dose rate scheme.

In 2011, Park [14] and colleagues conducted the first preclinical study to assess the effectiveness of PLDR, using advanced imaging techniques like micro-positron emission tomography/computed tomography (microPET/CT). They established glioblastoma multiforme (GBM) tumors in nude mice and treated them with either the standard daily radiation dose of 2 Gy or with PLDR, administered in ten 0.2 Gy pulses at 3 min intervals, totaling 14 Gy. The study tracked tumor growth weekly using microPET/CT imaging and confirmed findings with necropsy histopathology. They also evaluated damage to normal brain tissue by counting dead neural cells in sections of irradiated areas. The results showed a strong correlation between tumor size measured by CT and histopathology. Tumors treated with PLDR took significantly longer to grow compared to those treated with conventional radiation. Furthermore, PLDR resulted in fewer deaths of normal neural cells. Their study suggested that PLDR is more effective than standard radiation therapy in controlling tumor growth while causing less damage to normal tissues.

In another study [15], it was shown that PLDR might enhance the release of tumor antigens, which may amplify the adaptive immune response by promoting the expansion of the tumor-specific T-cell receptor repertoire, increasing the production of high-affinity tumor-specific antibodies, and supporting the generation of memory lymphocytes. These effects may contribute to an improved immune-mediated control of systemic disease.

Recently, Dos-Santos et al. [16] investigated PLDR’s effects at varying dose rates on two human cell lines, A549 and PC3, but found no significant differences in survival fractions. This supports PLDR’s clinical promise due to its tumor control and tissue-sparing at decreased dose rates. PLDR shows potential for treating large or recurrent cancers with limited normal tissue tolerance.

Zhang et al. [17] investigated the local tumor control efficacy of pulsed low dose rate radiotherapy (PLDR) for recurrent lung cancer and demonstrated that both conventional radiotherapy (RT) and PLDR significantly inhibited the growth of A549 xenografts compared to the control group. Similarly, Wang et al. [18] examined the in vivo tumor control efficacy of PLDR for prostate cancer treatment. Their results showed that tumors in both the PLDR and RT groups exhibited significant growth delays compared to the control group. However, there was no statistically significant difference in tumor control between the PLDR and conventional RT groups.

While a significant amount of research has been conducted on the effects of PLDR on tumor suppression in both lab settings and live subjects [17,19,20,21,22,23,24,25], there is a lack of studies examining its impact on normal tissue in vivo in animals. Therefore, this study primarily focuses on assessing the ability of PLDR to spare healthy tissues in a live animal setting, aiming to provide a clearer understanding of its overall safety and effectiveness in real-world therapeutic applications.

To achieve this, we initially performed a total-body irradiation (TBI) dose-gradation single-fraction study with immunocompetent C57BL/6 mice divided into three groups: control (0 Gy), PLDR, and conventional radiotherapy (CRT). Each group had two sub-groups with 3- and 5-day post-irradiation endpoints, with mice receiving an integral dose of 4, 6, 8, 10, and 12 Gy. Based on the results of the histological analysis of the spleen, lymph nodes, stomach, small intestine, large intestine, bone marrow, brain, pancreas, lungs, and heart, we proceeded to the second phase of targeted treatment. The findings from the first phase showed a particularly notable difference in the morphology of abdominal organs between CRT and PLDR treatments, which guided our approach to the second phase, which was focused on targeted treatment of abdominal organs.

The first challenge encountered was that, although tissue damage was graded by an independent pathologist in the initial phase, histopathological assessment could still vary between observers. This variability introduced a degree of subjectivity in distinguishing the effects of PLDR and CRT. To address this, we aimed to translate these findings into more objective, quantitative measures by focusing on post-treatment weight changes and survival.

The second challenge involved ethical considerations and limited resources, which necessitated minimizing the number of animals used in the experiment while still ensuring reliable results. To address this, we employed the $D_{100}$ value (the dose at which 100% of mice die) as a key metric. This approach was chosen to reduce uncertainty associated with small sample sizes and weight variability, despite efforts to ensure that mice in both the CRT and PLDR groups had comparable baseline weights. In contrast, using $D_{50}$ or other dose-response measures could have been disproportionately affected by random or unexpected deaths in small cohorts, thereby compromising the robustness and reproducibility of the findings.

After determining the fatal dose for single-fraction CRT settings, we administered the same dose under PLDR conditions and closely monitored the survival rates and body weight fluctuations in mice exposed to these two radiation therapy methods.

Subsequently, we replicated the experiment, collected histological samples of abdominal organs, and compared the organ damage differences between the two settings when a dose equivalent to the fatal dose for CRT was administered. In the final step, using a dose escalation scheme, we investigated the fatal dose for PLDR.

## 2. Materials and Methods

All procedures in studies involving animal experiments were in accordance with the ethical standards of the Institutional Animal Care and Use Committee (IACUC) and the Laboratory Animal Facility of Fox Chase Cancer Center.

### 2.1. Study Design and Test Subjects

#### 2.1.1. Phase I

In this study, forty-nine C57BL/6 mice were divided into three groups: a control group (9 mice receiving 0 Gy), and two experimental groups undergoing PLDR (*n* = 20) and CRT (*n* = 20) radiation treatments. Each of these experimental groups was further split into sub-groups, designated for analysis at either 3 or 5 days post-irradiation. These sub-groups included duplicates of mice that received total body irradiation of integral doses of 4, 6, 8, 10, and 12 Gy.

For histological examination, various organs and tissues were harvested, including the spleen, lymph nodes, stomach, small intestine, large intestine, bone marrow, brain, pancreas, lungs, and heart. These collected specimens were then stained using Hematoxylin and Eosin (H&E). The stained tissues underwent histopathological evaluation by a pathologist, who performed detailed scoring based on the observed morphological changes.

#### 2.1.2. Phase II.1, II.2, and II.3

In **phase II.1.1,** to establish the fatal dose threshold for whole-abdominal irradiation, we used a radiation dose escalation scheme. Mice were initially assigned to seven groups, each receiving a single-fraction whole-abdominal CRT dose of 14 Gy, 16 Gy, 17 Gy, 18 Gy, 19 Gy, 20 Gy, or 22 Gy (*n* = 2 per group). Survival was monitored daily, and body weight was recorded to aid in identifying the fatal abdominal dose. Following this initial assessment, additional mice were treated with doses near the suspected threshold to validate the findings. This brought the total number of mice to 26, distributed as follows: 14 Gy (*n* = 2), 16 Gy (*n* = 4), 17 Gy (*n* = 5), 18 Gy (*n* = 5), 19 Gy (*n* = 4), 20 Gy (*n* = 2), and 22 Gy (*n* = 4).

In **phase II.2**, ten C57BL/6 mice were divided into 2 groups of 5 mice receiving a single fraction of 18 Gy of PLDR or CRT whole-abdominal treatment. Survival and weight loss were monitored daily to compare the two treatments. The mice were euthanized at their respective 1, 3, 5, 7, and 9 days post-irradiation time points. For histological examination, various organs and tissues from the abdomen were harvested, including the stomach, small intestine, and large intestine, as well as bone marrow. These collected specimens were then stained using H&E. The stained tissues underwent histopathological evaluation by a pathologist, who performed detailed scoring based on the observed morphological changes.

Finally, in **phase II.3,** thirty-six C57BL/6 mice were divided into 10 groups of mice receiving a dose of 19, 20, 21, 22, 24, 26, 28, 29, 30, and 32 Gy of PLDR or CRT whole-abdominal treatment. Survival and weight loss were monitored daily to compare the two treatments.

### 2.2. Treatment and Setup

**Figure 1:**
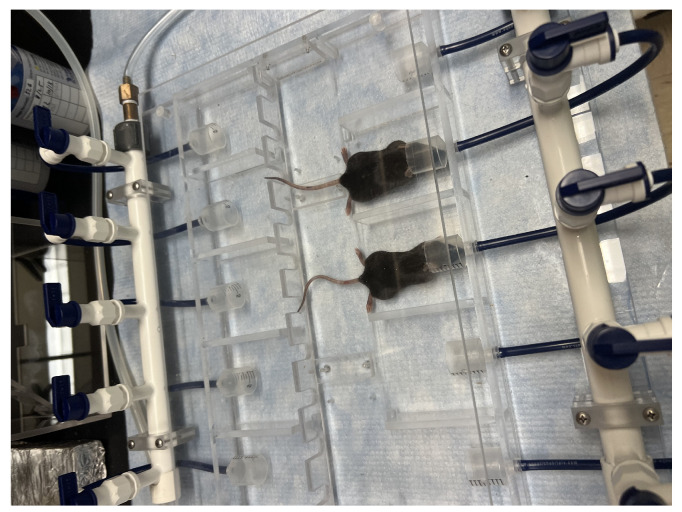
Setup for PLDR treatment of mice.

Phase I: Total body irradiation was delivered on a Varian Clinac iX accelerator with SSD (Varian, Palo Alto, CA, USA) setup of 100 cm with field size 40 cm × 40 cm, placing the central portion of the mice at the dmax for 6 MV photons with appropriate buildup (1 cm of boluse). The effective PLDR dose rate was 10 cGy/min and was achieved by delivering a train of 30 cGy pulses (subfractions) at 3 min intervals. CRT utilized a clinically relevant dose rate of 500 MU/min. Figure 1 Provides an overview of the treatment setup.

Phase II.1, II.2, and II.3: Whole-abdomen treatment delivery was performed on Varian Clinac iX accelerator with SSD setup of 100 cm and 6MV photon with appropriate buildup (1 cm of bolus) using a treatment field of 2.2 cm × 40 cm. The effective PLDR dose rate was 6.6 cGy/min and was achieved by delivering a train of 20 cGy pulses at 3 min intervals. CRT utilized a clinically relevant dose rate of 500 MU/min. During the treatment, mice were anesthetized with an inhalation of 2–3% isoflurane in oxygen.

## 3. Results

### 3.1. Treatment and Setup

#### 3.1.1. Phase I

**Figure 2:**
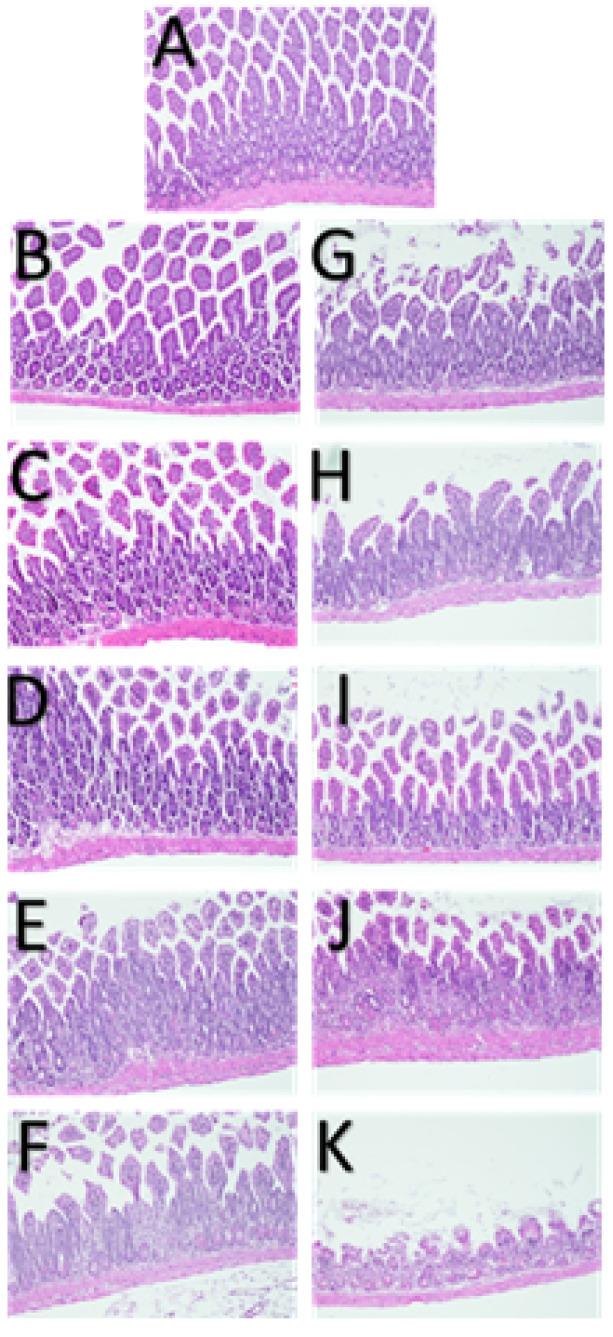
Histology of small intestine 3 days after C57BL/6J mice were TBI-treated. Typical tissue damage was observed in response to radiation in a dose-dependent manner. (**A**) Control (top center); (**B**–**F**) PLDR (left column); (**G**–**K**) CRT (right column). Marked increase in severity of atrophy and adenomatous hyperplasia was observed in CRT groups for the same dose when compared to PLDR. H&E staining; magnification ×100.

The histological staining of tissue slides revealed that both conventional and pulsed low dose rate radiotherapy led to a dose-dependent escalation in atrophy and adenomatous hyperplasia compared to the control group. This effect was particularly evident in the small intestine, as depicted in Figure 2. Furthermore, independent histological assessments conducted by a pathologist underscored notably more severe tissue damage in the small intestine of mice subjected to CRT at every integral dose level. In contrast, those treated with PLDR exhibited comparatively less damage. This distinction underscores the tissue-sparing advantage of the PLDR method in the small intestine.

**Figure 3:**
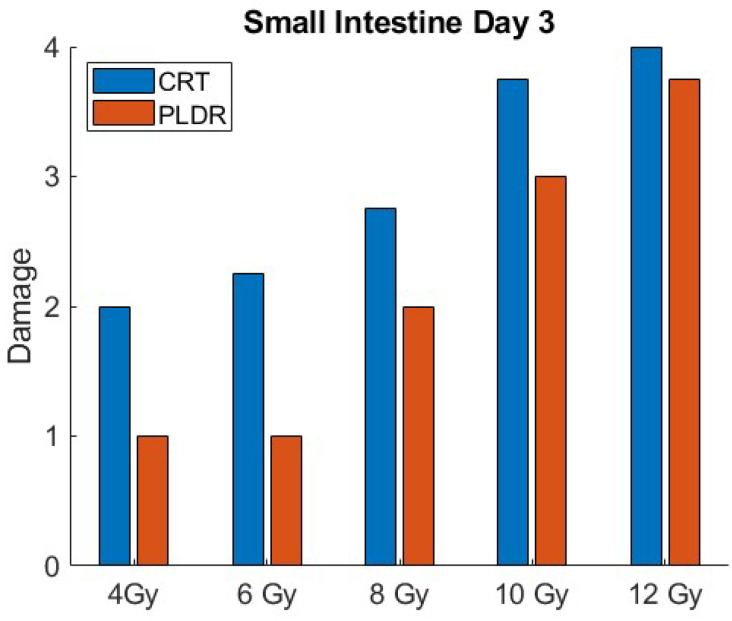
Small intestine tissue toxicity for different doses of CRT and PLDR at day 3.

**Figure 4:**
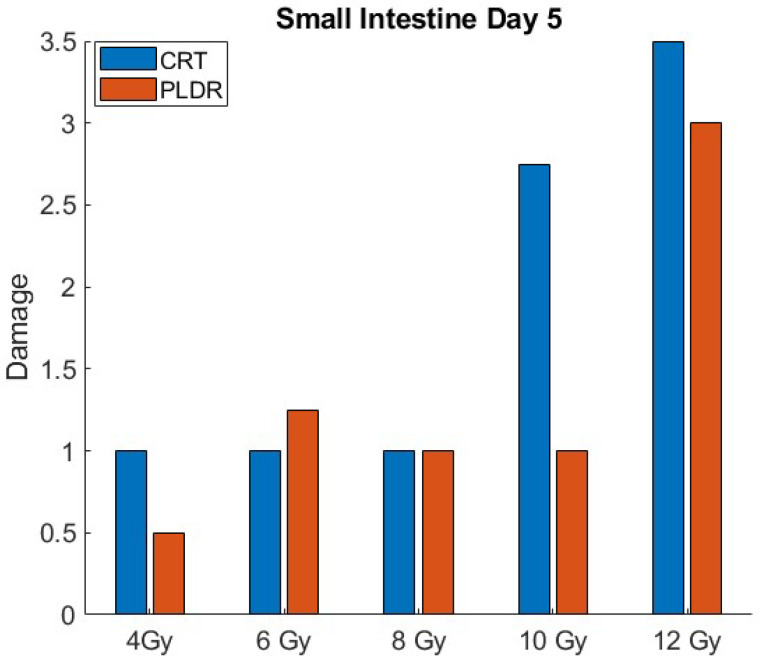
Small intestinetissue toxicity for different doses of CRT and PLDR at day 5.

Figure 3 and Figure 4 illustrates the quantitative differences in radiation-induced toxicity (damage) in the small intestine at day 3 and 5 for both PLDR and CRT. Tissue toxicity is graded based on H&E histopathologic score, with the score of 4 representing the highest level of tissue damage.

#### 3.1.2. Phase II.1

**Figure 5:**
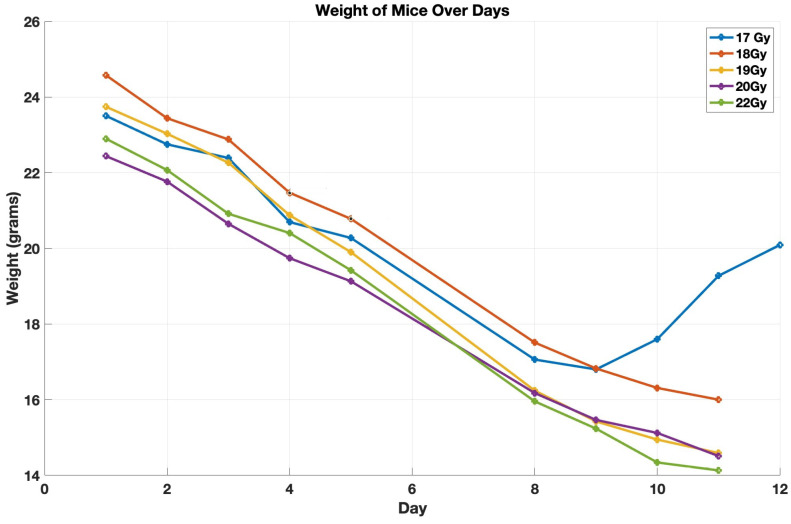
The average body weight of the mice receiving different doses of CRT.

**Figure A1:**
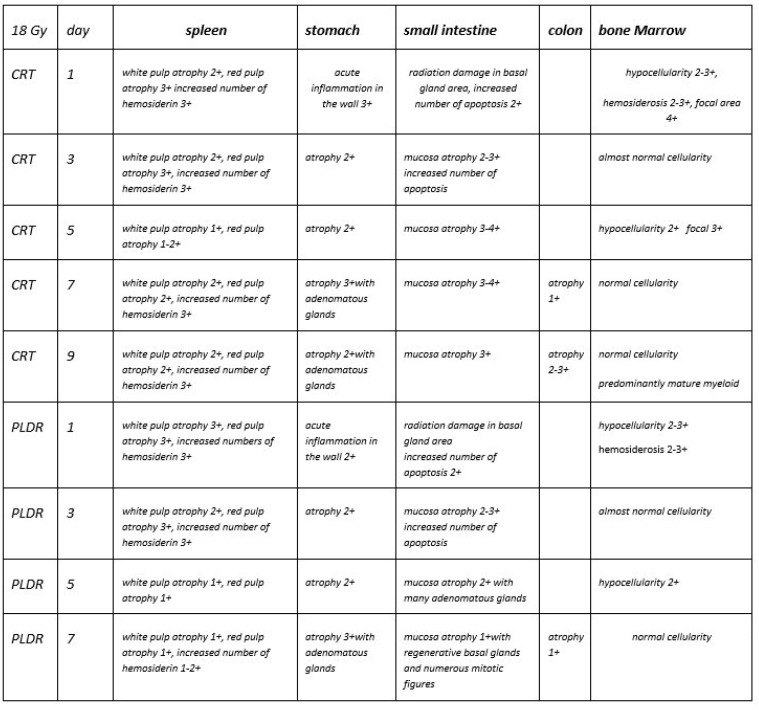
Histological changes inspleen, lymph nodes, stomach, small intestine, large intestine and bone marrow 3 and 5 days after irradiation with CRT or PLDR, scored from 1–4—higher number = greater the tissue damage.

After administering the designated radiation doses, we tracked the weight and survival of the mice. In groups receiving up to 17 Gy, an initial weight loss was observed, but these mice started regaining weight after 10 days and ultimately survived. In contrast, the groups exposed to 18 Gy or higher of CRT demonstrated a persistent decrease in weight, leading to their eventual death. The weight loss for each group for the whole-abdomen treatment for doses 17 Gy and higher is detailed in Figure 5. The outcomes of this experiment of CRT indicate that 18 Gy represents the $D_{100}$, the dose level at which all subjects died. Figure A1 in Appendix A shows the detailed histological report.

#### 3.1.3. Phase II.2

**Figure 6:**
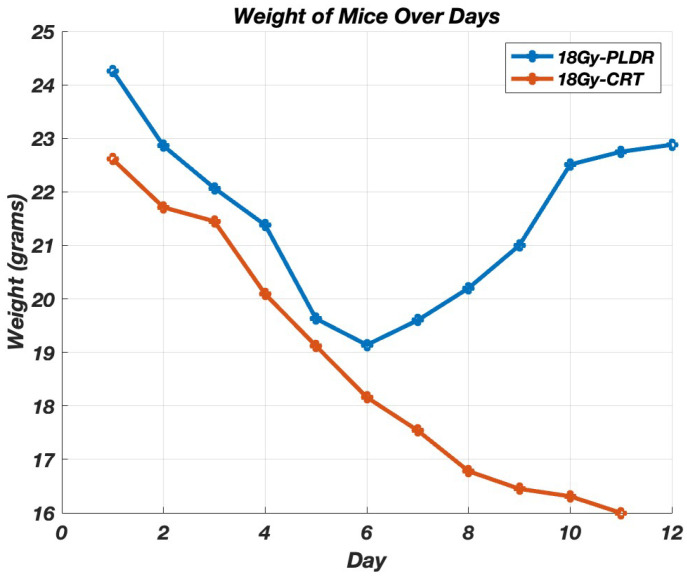
The average body weight of the mice receiving 18 Gy of CRT (*n* = 5) or PLDR (*n* = 5) treatment.

**Figure 7:**
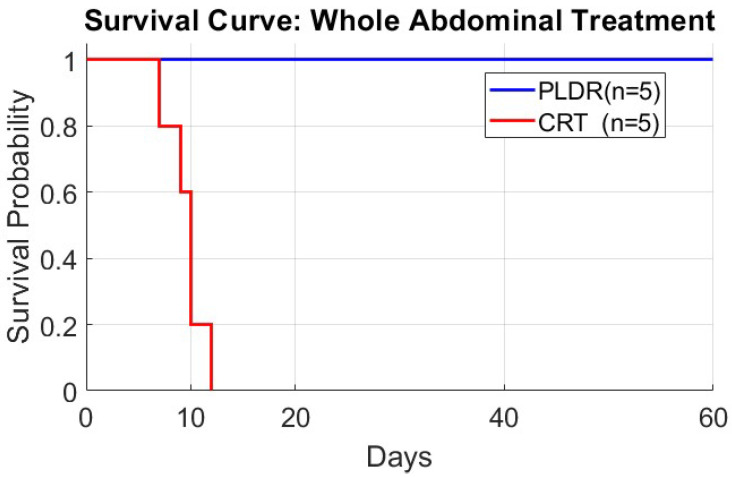
Survival curve of whole-abdominal 18 Gy treatment for PLDR (*n* = 5) and CRT (*n* = 5).

**Figure 8:**
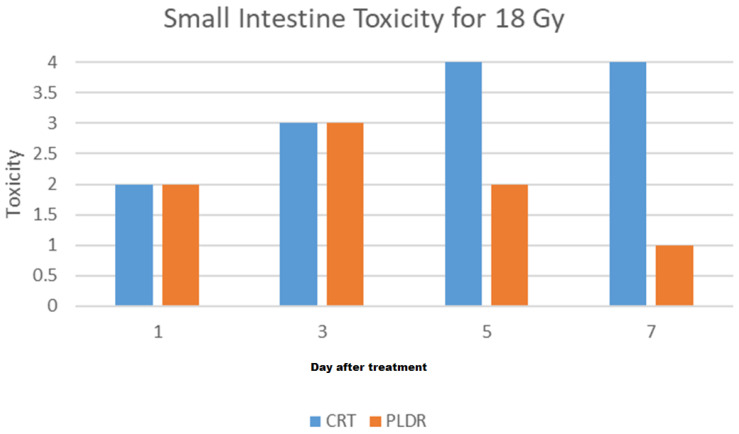
Small intestine tissue toxicity for 18 Gy of PLDR and CRT 1, 3, 5, and 7 days after treatment.

**Figure A2:**
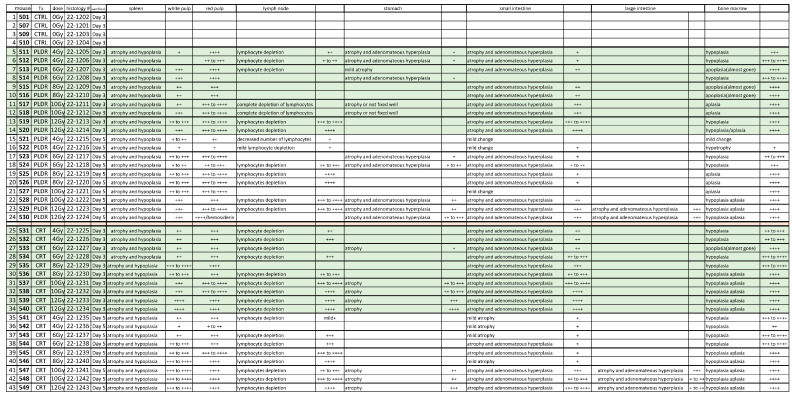
Histological assessment of tissue toxicity was performed using a standardized hematoxylin and eosin (H&E) histopathologic scoring system, where a score of 4 indicates the highest degree of tissue damage. Grading was conducted selectively across relevant tissues, with higher scores corresponding to more extensive histopathological injury. Observed features included subchronic inflammatory cellular infiltrates and adenomatous basal gland proliferation, accompanied by minimal mitotic activity.

Since we determined 18 Gy as the fatal dose, we delivered it using two techniques, each group containing five mice. Initially, we just monitored the body weight and survival rate (Figure 6 and Figure 7). We replicated the experiment, but this time the mice were euthanized at day 1, 3, 5, 7, and 9. Organ samples were harvested and graded by a pathologist. Figure 8 shows the results of the histological grading for the small intestine, where a higher grade indicates more damage, showing that tissues with sub-chronic inflammatory cellular infiltrates and adenomatous basal glands exhibit low levels of mitosis. Figure A2 in Appendix A shows the detailed histological report. The results show that although the initial degree of toxicity is the same in both PLDR and CRT, the PLDR group begins to recover after day 5, whereas tissue toxicity continues to increase in the CRT group.

#### 3.1.4. Phase III

In Phase 3, we performed a dose escalation scheme to find the fatal dose for whole-abdominal PLDR radiation treatment. The body weights were recorded after the treatment with 19, 20, 21, 24, 26, 28, 29, 30, 32, and 34 Gy. The results showed that 29 Gy was the fatal dose for PLDR whole-abdominal treatment.

## 4. Discussion

Our research proposes that pulsed low dose rate radiotherapy (PLDR) may offer similar tumor control to conventional radiation therapy (CRT) with the added advantage of sparing normal tissues and reducing toxicity. Previous in vitro and in vivo studies by our group have already established PLDR’s efficacy in tumor control. Building on this, our current study explores PLDR’s potential in reducing damage to normal tissues.

The study was structured into three distinct phases. The initial phase involved treating healthy mice with a progressively increasing dose of radiation, to compare the extent of organ damage caused by PLDR and CRT. In particular, the results of this phase indicated that PLDR causes significantly less harm to normal tissues, a difference that was especially marked in the abdominal region.

Encouraged by these findings, the second phase focused specifically on abdominal treatment, assessing parameters such as weight loss, fatal radiation dose, and survival rates in mice subjected to whole-abdominal irradiation. Our approach began by determining the fatal dose for CRT in abdominal irradiation, which was identified as 18 Gy. Repeating this dose with PLDR, we observed marked differences: notably, while 18 Gy was fatal under CRT, it was not under PLDR. Furthermore, weight loss trends differed between the groups—both experienced initial weight loss, but the decline was less severe in the PLDR group, and these mice gradually regained weight. Histological examination of abdominal tissues also revealed less damage in the PLDR-treated group, with signs of tissue repair evident by the ninth day.

The final phase of our study determined the lethal dose for PLDR, which was higher than that for CRT, at 29 Gy. This finding suggests that PLDR requires a dose more than 60% greater than CRT to produce a comparable level of organ damage, reinforcing the notion that PLDR is significantly less toxic to abdominal organs.

Using the concept of EQD2 and assuming a single-fraction treatment with an α/β ratio of 3—appropriate for modeling normal tissue response—the fatal dose equates to 75.6 Gy for CRT and 185.6 Gy for PLDR, which is more than double. This implies that, in clinical practice, one could deliver 46 fractions of 2 Gy using PLDR instead of 21 fractions of 2 Gy with CRT. Such a difference could be critical in achieving tumor control while minimizing toxicity to organs like the liver.

These results can also be useful for re-treatment; instead of treating a recurrent cancer with a CRT scheme, which limits the physician’s ability to prescribe enough dose to control the cancer due to organ-specific dose limits, one can use PLDR, which is less toxic and has a higher dose limit.

One important point to emphasize is that this study focuses solely on the short-term effects and comparisons between PLDR and CRT. Future studies investigating long-term functional outcomes of PLDR are indeed a valuable direction for research. However, even in the absence of detailed long-term data, our results suggest a degree of tissue recovery and tolerability, supporting the potential of PLDR in enhancing tissue sparing, despite not assessing long-term functional outcomes in this study.

Importantly, the study was designed to compare single-fraction CRT and single-fraction PLDR. While it focuses on single-fraction regimens, we believe the findings are also relevant to conventional fractionation, given well-established radiobiological principles—particularly the concept of EQD2. Therefore, with appropriate consideration of dose equivalence, these results may be extrapolated to conventional multi-fraction settings.

## 5. Conclusions

Pulsed low dose rate radiotherapy (PLDR) is a novel technique that promises equivalent tumor control compared to conventional radiotherapy (CRT), while potentially reducing normal tissue toxicity. In this study, we investigated the short-term acute toxicity of PLDR on normal tissue in comparison to CRT. Our results demonstrate that PLDR is associated with improved short-term acute toxicity profiles. Although the assessment of long-term functional toxicity in normal tissue following PLDR remains to be explored, the short-term findings support enhanced normal tissue tolerability and recovery, even in the absence of comprehensive long-term outcome data.

## Data Availability

The original contributions presented in this study are included in the article.Further inquiries can be directed to the corresponding author.
